# Off-the-shelf barrier for emergency intubation in the cardiac catheterization laboratory during the coronavirus disease 2019 (COVID-19) pandemic

**DOI:** 10.1007/s00392-020-01696-9

**Published:** 2020-07-04

**Authors:** Bruno Scheller, Davor Vukadinovic, Sebastian Ewen, Felix Mahfoud

**Affiliations:** 1 Internal Medicine III, Cardiology, Angiology and Intensive Care Medicine, Saarland University Hospital, Saarland University, Homburg, Saar Germany; 2 Clinical and Experimental Interventional Cardiology, University of Saarland, Homburg, Saar Germany

**Keywords:** COVID-19, Endotracheal intubation, Catheterization laboratory

## Abstract

With the spread of SARS-CoV-2, it is expected that cases of acute coronary syndrome in the setting of coronavirus disease 2019 (COVID-19) develop. As expensive and sophisticated protection devices are not widely available, we have been working on a simple, off-the-shelf protection device for endotracheal intubation of potentially infected patients. For this purpose, we used a large transparent plastic bag (such as the sterile protective cover of the lead glass shield) for protection from airborne infections. The cover is moved over the patient's head from cranial to caudal, covering the catheter table including the torso with no need for patient mobilization. The intubation is done conventionally under direct visual control.

**Electronic supplementary material:**

The online version of this article (10.1007/s00392-020-01696-9) contains supplementary material, which is available to authorized users.

## Introduction

As the SARS-CoV-2 virus continues to infect patients with cardiovascular disease, it is expected that cases of acute coronary syndrome (ACS) together with coronavirus disease 2019 (COVID-19) occur [1, 3]. In patients with ST-segment elevation myocardial infarction (STEMI), it is recommended to immediately perform primary coronary intervention. STEMI can be complicated by cardiogenic shock requiring endotracheal intubation and mechanical ventilation also during treatment in the catheterization laboratory. Given the logistical challenges, at this point, in the majority of patients, no information on COVID-19 infection will be available. While for intensive care units, extensive preparatory and protective measures have been suggested aiming at reducing the aerosol contamination and, above all, to protect the personnel performing or assisting the laryngoscopy and endotracheal intubation [2], no such recommendations exist for cardiac catherization laboratories.

## Case report

In emergency situations during cardiac catherization, there may be limited human resources and time to apply extensive protective measures. Against this background, we present a simple, off-the-shelf protective measure for emergency endotracheal intubation in the cardiac catheterization laboratory, using the sterile protective cover of the lead glass shield, deflected from its normal purpose.

**Fig. 1:**
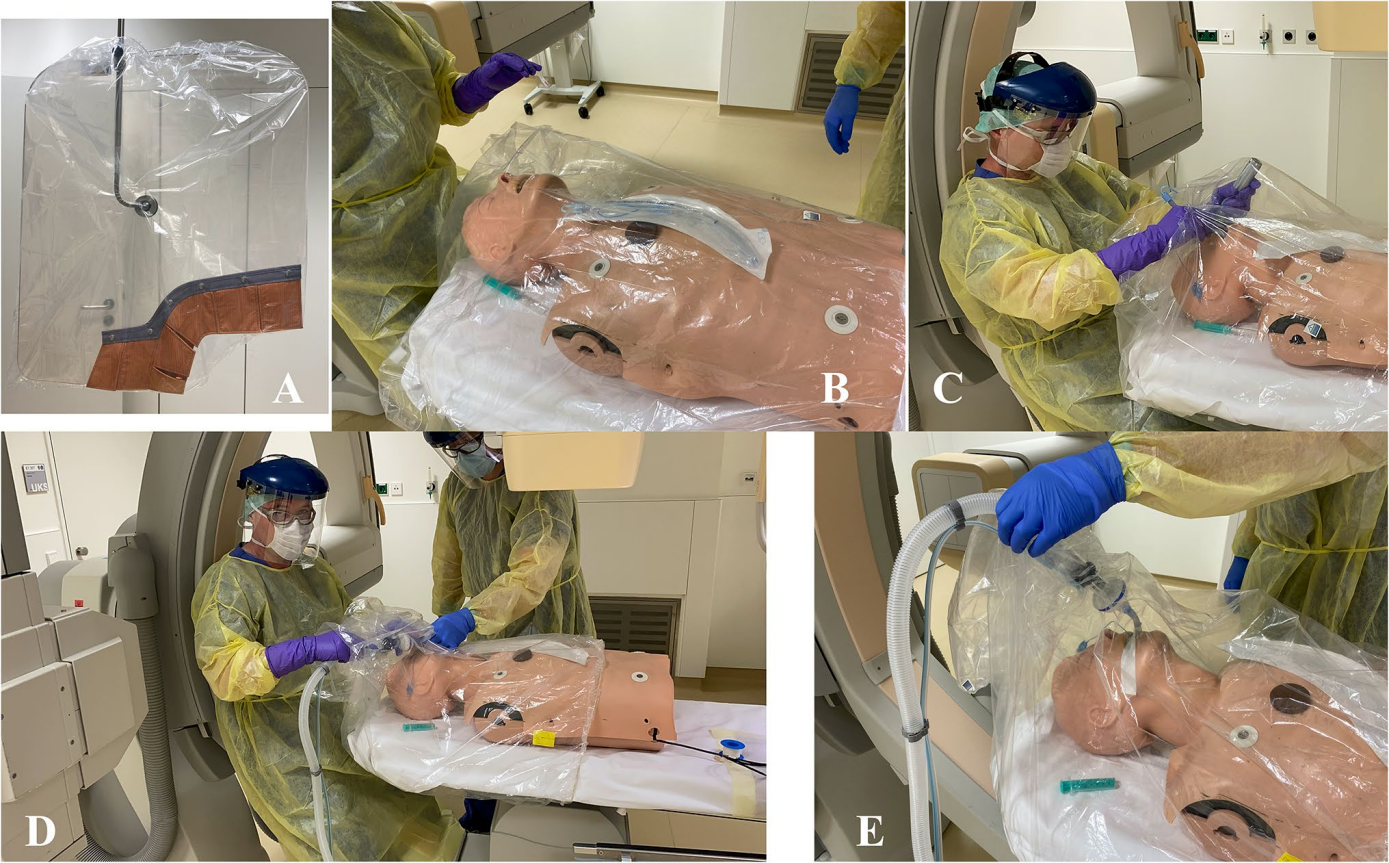
Endotracheal intubation in the cardiac catheterization laboratory using a simple, off-the-shelf protective device. **a** Sterile protective cover mounted on the lead glass shield. **b** The cover is moved from cranial to caudal over the patient’s head, covering the catheter table so that the patient is not mobilized. The tight rubber band of the cover provides good insulation of the abdominal area. **c** Intubation is done conventionally under direct visual control. **d**, **e** Blocking of the tube, connection to the ventilator with breathing filter, and fixation of the tube are also carried out by the intubating physician with assistance from outside

For this, two slots are cut in the cover for the hands of the physician performing the endotracheal intubation. The cover is moved over the patient's head from cranial to caudal, covering the catheter table including the torso with no need for patient mobilization. The tight rubber band of the cover provides good insulation of the abdominal area. The intubation is done conventionally under direct visual control through the transparent sheet or if available by video laryngoscope. Blocking of the tube, connection to the ventilator with breathing filter and fixation of the tube are carried out by the intubating physician with assistance from outside (Fig. 1). The cover can be left in place to prevent contamination of the room. The procedure is shown in detail in the three online videos (covering the patient, endotracheal intubation, and connection of ventilator).

## Discussion

With the spread of SARS-CoV-2, it is expected that cases of acute coronary syndrome in the setting of coronavirus disease 2019 (COVID-19) develop, which will pose major challenges to cath lab staff around the globe. As expensive and sophisticated protection devices are not widely available, we have been working on a simple, off-the-shelf protection device for endotracheal intubation of (potentially) infected patients. This measure may indeed turn out to be helpful during the pandemic. Furthermore, this concept could also be adopted in other emergency situations outside the catheter laboratory.

## Electronic supplementary material

Below is the link to the electronic supplementary material.

Supplementary file1 Online Video 1: Covering the patient with the sterile protective cover. The cover is moved from cranial to caudal over the patient's head, covering the catheter table so that the patient is not mobilized. The tight rubber band of the cover provides good insulation of the abdominal area. (MOV 84044 kb)

Supplementary file2 Online Video 2: Intubation is done conventionally under direct visual control. (MOV 170474 kb)

Supplementary file3 Online Video 3: Blocking of the tube, connection to the ventilator with breathing filter and fixation of the tube are also carried out by the intubating physician with assistance from outside. (MOV 41918 kb)
