# Correction to: Off‑the‑shelf barrier for emergency intubation in the cardiac catheterization laboratory during the coronavirus disease 2019 (COVID‑19) pandemic

**DOI:** 10.1007/s00392-020-01713-x

**Published:** 2020-08-12

**Authors:** Bruno Scheller, Davor Vukadinovic, Andreas Link, Sebastian Ewen, Felix Mahfoud

**Affiliations:** 1 Internal Medicine III, Cardiology, Angiology and Intensive Care Medicine, Saarland University Hospital, Saarland University, Homburg, Saar Germany; 2 Clinical and Experimental Interventional Cardiology, University of Saarland, Homburg, Saar Germany

## Correction to: Clinical Research in Cardiology 10.1007/s00392-020-01696-9

During submission the author name Andreas Link was unfortunately omitted. The correct author list reads as follows:

Bruno Scheller^1,2^, Davor Vukadinovic^1^, Andreas Link^1^, Sebastian Ewen^1^, Felix Mahfoud^1^

^1^Internal Medicine III, Cardiology, Angiology and Intensive Care Medicine, Saarland University Hospital, Saarland University, Homburg, Saar, Germany

^2^Clinical and Experimental Interventional Cardiology, University of Saarland, Homburg, Saar, Germany

